# Preclinical Characterization of a Novel Monoclonal Antibody NEO-201 for the Treatment of Human Carcinomas

**DOI:** 10.3389/fimmu.2017.01899

**Published:** 2018-01-04

**Authors:** Massimo Fantini, Justin M. David, Olga Saric, Alexander Dubeykovskiy, Yongzhi Cui, Sharon A. Mavroukakis, Andrew Bristol, Christina M. Annunziata, Kwong Y. Tsang, Philip M. Arlen

**Affiliations:** ^1^Precision Biologics, Inc., Rockville, MD, United States; ^2^Synthetic Biologics, Inc., Rockville, MD, United States; ^3^Women’s Malignancy Branch, Center for Cancer Research, National Cancer Institute, National Institutes of Health, Bethesda, MD, United States

**Keywords:** monoclonal antibody, tumor-associated antigen, antibody-dependent cellular cytotoxicity, complement-dependent cytotoxicity, natural killer cell

## Abstract

NEO-201 is a novel humanized IgG1 monoclonal antibody that was derived from an immunogenic preparation of tumor-associated antigens from pooled allogeneic colon tumor tissue extracts. It was found to react against a variety of cultured human carcinoma cell lines and was highly reactive against the majority of tumor tissues from many different carcinomas, including colon, pancreatic, stomach, lung, and breast cancers. NEO-201 also exhibited tumor specificity, as the majority of normal tissues were not recognized by this antibody. Functional assays revealed that treatment with NEO-201 is capable of mediating both antibody-dependent cellular cytotoxicity (ADCC) and complement-dependent cytotoxicity (CDC) against tumor cells. Furthermore, the growth of human pancreatic xenograft tumors *in vivo* was largely attenuated by treatment with NEO-201 both alone and in combination with human peripheral blood mononuclear cells as an effector cell source for ADCC. *In vivo* biodistribution studies in human tumor xenograft-bearing mice revealed that NEO-201 preferentially accumulates in the tumor but not organ tissue. Finally, a single-dose toxicity study in non-human primates demonstrated safety and tolerability of NEO-201, as a transient decrease in circulating neutrophils was the only related adverse effect observed. These findings indicate that NEO-201 warrants clinical testing as both a novel diagnostic and therapeutic agent for the treatment of a broad variety of carcinomas.

## Introduction

Cancer represents one of the most frequent causes of mortality worldwide, with an estimated 20 million new cases expected annually as early as 2025 (1). Conventional methods of treating cancer, such as surgery, radiation, and chemotherapy, often elicit severe side-effects yet fail to cure the majority of patients with advanced disease, leading to relapse (2). More recent treatment modalities have been developed to selectively target cancerous cells while largely sparing normal healthy tissues. Among them, immunotherapy has become an important treatment option for cancer patients as it revolutionizes the field of cancer medicine.

An underlying principle of cancer immunotherapy is known as immunoediting (3), which is an extrinsic mechanism of cancer suppression that initiates only after cellular transformation has occurred and intrinsic mechanisms of cancer suppression have failed. The immunoediting process occurs in three phases; elimination, equilibrium, and escape. During the elimination and equilibrium phases, respectively, immune rejection of cancer cells either predominates or balances with cancer cell proliferation to control malignant growth. In the escape phase, however, cancer cells once held in check may escape immune recognition due to insensitivity to immune effector mechanisms and/or induction of immune suppression in the tumor microenvironment. Cancer cells that escape immune recognition are then able to more freely proliferate and grow into clinically apparent disease (4). The aim of cancer immunotherapy is to keep cancer cells in the elimination and/or equilibrium phase by generating and/or amplifying antitumor immune responses to counteract tumor growth, delay tumor recurrence, and prolong survival (5–8). Common approaches include treating patients with checkpoint inhibitory antibodies, antitumor vaccines, and chimeric antigen receptor-T cells, all of which leverage adaptive immunity by T cells. However, innate immunity can also generate and potentiate antitumor responses, and tumor-targeting monoclonal antibodies (mAbs) can be used to stimulate innate antitumor immunity (9).

NEO-201 is a novel humanized IgG1 mAb that was generated against the Hollinshead allogeneic colorectal cancer vaccine platform (10, 11). The immunogenic components of this vaccine were tumor-associated antigens (TAAs) that were derived from tumor membrane fractions pooled from surgically resected specimens from 79 patients with colon cancer (12). These membrane fractions were semi-purified, screened for delayed-type hypersensitivity in colon cancer patients versus healthy volunteers, and evaluated in clinical trials in patients with refractory colorectal cancer (12–14). These trials reported clinical benefit as defined by both antitumor response and significant prolongation in overall survival in patients who developed a sustained IgG response in addition to a cell-mediated response against the vaccine, thereby suggesting that the vaccine contained immunogenic components capable of generating antitumor antibodies (15). This original colorectal cancer vaccine was used to generate monoclonal antibodies in mice, yielding the previously described ensituximab (NPC-1C/NEO-102) (16–19) and NEO-201. Preliminary investigation indicates that NEO-201 may bind tumor-associated variants of CEACAM family members (20), and efforts are underway to further characterize the antigen(s) and specific epitope(s) recognized by NEO-201.

Monoclonal antibodies consist of a unique antigen-binding region (fragment antigen-binding, Fab) that is specific to a given mAb, and a constant region (fragment crystallizable, Fc) that is common to all mAbs of the same isotype. The Fc region is capable of modulating immune cell activity by engaging with Fc receptor family members expressed on the surface of specific immune cell types. In particular, human IgG1 mAbs can interact with Fc gamma receptor IIIa (FcγRIIIa, CD16) expressed on macrophages and natural killer (NK) cells. This interaction can stimulate macrophages to phagocytose mAb-opsonized cancer cells and can activate NK cells to degranulate and lyse cancer cells through a mechanism known as antibody-dependent cellular cytotoxicity (ADCC). ADCC has been shown to be a key mediator of antitumor effects *in vivo* in many preclinical studies and plays an important role in the mechanism of action of several mAbs used for cancer therapy (21). Examples of clinically approved mAbs, that can mediate ADCC, include trastuzumab, which targets the HER2 receptor for breast cancer (21, 22); rituximab, which targets the pan-B-cell marker CD20 for lymphoma (21, 23); cetuximab, which targets the epidermal growth factor receptor for colorectal and head and neck cancer (21, 24–26); and avelumab, which targets the immunosuppressive ligand PD-L1 for Merkel cell carcinoma and bladder cancer (27). Additionally, the Fc region can also interact with the C1 complex to activate complement-dependent cytotoxicity (CDC), in which a proteolytic cascade culminates in the formation of pores in the plasma membrane that cause the lysis of cells targeted by the antibody. Antitumor CDC can be readily demonstrated *in vitro*, but whether it is crucial for the clinical efficacy of mAb therapy in cancer remains controversial (28).

This study was undertaken to assess the *in vitro* binding characteristics and *in vivo* activity and localization of NEO-201 in preclinical models in preparation for assessing its safety and efficacy in clinical trials. NEO-201 exhibited broad reactivity against a range of human carcinoma cell lines and tumor tissues, but was not observed to bind the majority of healthy tissues. In addition, NEO-201 exhibited both ADCC and CDC activity against human carcinoma cells *in vitro* and largely attenuated the growth of human pancreatic xenograft tumors *in vivo* both alone and in combination with human peripheral blood mononuclear cells (PBMCs) as the effector cell source for ADCC. Finally, a single-dose toxicity study in non-human primates demonstrated safety and tolerability of NEO-201, as a transient decrease in circulating neutrophils was the only adverse effect observed. These studies provide the rationale for the potential clinical utility of NEO-201 as a novel therapeutic agent for the treatment of a wide variety of solid tumors.

## Materials and Methods

### Cell Lines and Culture

The following human carcinoma cell lines were obtained from the American Type Culture Collection (Manassas, VA, USA): colon (COLO 205, HT-29, LS174T, SW1116, SW1463, SW480), pancreas (ASPC-1, BxPC-3, CAPAN-2, CFPAC-1, PANC-1), breast (AU-565, BT-474, BT-549, HCC1500, HCC1937, HCC38, MDA-MB-468, SK-BR-3, T-47D, ZR-75-1), and lung (CALU-1, H1703, H226, H441, H520, H522, HCC4006, HCC827, SK-LU-1). All cell cultures were maintained in RPMI 1640, DMEM, or IMDM culture medium (Corning, Corning, NY, USA) as designated by the provider for propagation and maintenance. Culture medium was supplemented with 10% USA-sourced and heat-inactivated HyClone Fetal Bovine Serum Defined (GE Healthcare Life Sciences, Issaquah, WA, USA), 100 U/mL penicillin, 100 µg/mL streptomycin (Corning Life Science, Manassas, VA, USA). PBMCs from healthy volunteer donors were obtained from the National Institutes of Health Clinical Center Blood Bank (NCT00001846) under the appropriate Institutional Review Board approval and informed consent.

### Generation of the Humanized NEO-201 mAb

The Hollinshead colon cancer-specific vaccine was used as the immunogenic material to generate monoclonal antibodies in mice. The method for the preparation of tumor-associated proteins and peptides has been previously described (13). In brief, cancer tissue was minced and used to generate a single cell suspension that was then subjected to hypotonic saline membrane extraction, a series of centrifugation steps, and followed with low frequency sonication. The resulting membrane-extracted proteins were fractionated on Sephadex G-200 resin or by electrophoretic methods, then concentrated and quantitated (10–12). The TAA preparation was admixed with complete Freund’s adjuvant and injected subcutaneously in BALB/c mice. This was followed by three booster injections in incomplete Freund’s adjuvant, separated by 2–3 weeks. Mouse serum was tested by ELISA for antibody responses against the immunizing antigen and mice with potent responses were used to generate immortalized hybridoma cells by fusing the mouse B cells from the spleen with the SP2/0-Ag14 myeloma cell line and selecting cells that grew and produced mouse immunoglobulins (IgGs). From these mouse IgGs, the murine 16C3 clone (m16C3) was chosen based upon reactivity with colon tumor cell membrane extract derived from LS174T or HT-29 cells as determined by ELISA. The cDNAs encoding the heavy and light chain IgG1 were determined from RNA isolated from hybridoma clone 16C3 E12 and shown to be unique (14). As described in the US patent 7829678, the m16C3 protein sequence was humanized as h16C3 and designated NEO-201. Humanization was performed *in silico* by replacing mouse sequences outside the complementarity-determining regions (CDRs) of the Fab region of both heavy and light chain proteins with human Fab sequences, and retaining the three mouse CDR sequences from each chain. The Fc regions of the heavy and light chains were selected from human IgG1 isotype used in other humanized approved mAb products. The amino acid sequence was back-translated to DNA, which was optimized for protein expression in CHO cells. The DNA for heavy and light chain h16C3 was then synthesized chemically, cloned into mammalian expression plasmids, and transfected into mammalian cell lines (HEK293T and CHO). Several stable CHO cell lines expressing recombinant h16C3 were derived and banked. Purified recombinant h16C3 was retested in studies which verified that the humanized 16C3 antibody had similar characteristics as the original m16C3 antibody (14).

### Flow Cytometry

Binding of NEO-201 to human carcinoma cell lines was analyzed by flow cytometry. Cells (1.0 × 10^6^) were incubated with 1 µL per test of LIVE/DEAD Fixable Aqua (Thermo Fisher Scientific, Waltham, MA, USA) in 1× phosphate buffered saline (PBS) for 30 min at 4°C to accomplish live versus dead cell discrimination. Cells were then centrifuged, washed twice with cold PBS, and then stained with Pacific Blue-conjugated NEO-201 antibody (BioLegend, San Diego, CA, USA) in 1× PBS + 1% BSA (Teknova, Hollister, CA, USA) for 30 min at 4°C. After staining, cells were washed twice with cold PBS and examined using a FACSVerse flow cytometer (BD Biosciences, San Jose, CA, USA). Analysis of cellular fluorescence was performed using BD FACSuite software (BD Biosciences, San Jose, CA, USA). Positivity was determined using fluorescence minus one controls. Staining values >10% positive were considered positive for NEO-201 expression. Positive cell lines were ranked according to their quantified expression level (% positive × MFI), and then sorted into groups of low (<200), medium (200–1,000), and high (>1,000) expression.

### Immunohistochemistry (IHC)

Tissue microarrays for colon samples (CO808, CO951) were obtained from US Biomax (Rockville, MD, USA), and AccuMax tissue microarrays for colon [A303(I)], pancreas [A207(II), A307], stomach (A209), lung [A206(V), A306], breast [A202(VI), A712], uterus (A212), ovary [A212, A213(II)], prostate [A302(IV)], and various normal [A103(VII)] samples were obtained from Accurate Chemical and Scientific Corporation (Westbury, NY, USA). NEO-201 was biotinylated using the Biotin Protein Labeling Kit (Roche, Basel, Switzerland) as per manufacturer’s instructions. Slides were baked at 60°C for 20 min, deparaffinized with xylene, and rehydrated with a graded ethanol series. Slides were then subjected to peroxide blocking using Peroxidazed I solution (Biocare Medical, Concord, CA, USA) for 2 min, avidin blocking using avidin solution (Biocare Medical, Concord, CA, USA) for 10 min, biotin blocking using biotin solution (Biocare Medica, Concord, CA, USA) for 10 min, and protein blocking using CAS-Block histochemical reagent (Thermo Fisher Scientific, Waltham, MA, USA) for 10 min. Slides were then incubated at room temperature with negative control biotinylated human IgG1 kappa (Ancell, Bayport, MN, USA) or biotinylated NEO-201 at 10 µg/mL diluted in 1× PBS for 2 h. Detection was enabled with Dako streptavidin–HRP conjugate (Agilent Technologies, Santa Clara, CA, USA) at 1:300 for 30 min, incubation with DAB peroxidase substrate (Thermo Fisher Scientific, Waltham, MA, USA) for 1–3 min, and counterstaining with hematoxylin. Each microarray tissue spot was evaluated by light microscopy for cell staining intensity using the following scale: 0 (negative), 1+ (weak), 2+ (moderate), 3+ (strong), 4+ (very strong). A tissue spot was recorded as positive if it contained cells stained with intensity ≥1+.

### ADCC Assay

Antibody-dependent cellular cytotoxicity assays were performed using a modification of a previously described procedure (27). Negative selection of NK cells from human donor PBMCs was performed using the EasySep Human NK Cell Isolation Kit (StemCell Technologies, Vancouver, BC, Canada) according to the manufacturer’s protocol. Purified NK cells were incubated overnight in RPMI-1640 medium supplemented with l-glutamine, 10% FBS, and antibiotics. On the day of the assay, target cells (CFPAC-1, ASPC-1) were labeled with 10 µM calcein AM cell-permeant dye (Thermo Fisher Scientific, Waltham, MA, USA) for 30 min and then seeded in triplicate at 3.0 × 10^3^ cells/well into black-walled flat-bottom 96-well culture plates (655090, Greiner Bio-One, Kremsmünster, Austria). Target cells were then treated with 10 µg/mL of human IgG1 isotype control antibody (Thermo Fisher Scientific, Waltham, MA, USA) or NEO-201 antibody unless otherwise indicated, and then NK cells were added at effector-to-target (E:T) ratios of 12.5:1 and 25:1. After 4 h incubation at 37°C, 1.67 µg/mL propidium iodide (PI) (Thermo Fisher Scientific, Waltham, MA, USA) was added to each well, the plate was imaged using the Celigo Imaging Cytometer (Nexcelom Bioscence LLC, Lawrence, MA, USA), and the numbers of live target cells (calcein AM+/PI−) versus dead cells (calcein AM+/PI+ or calcein AM−/PI+) was analyzed and recorded by the Celigo Imaging Cytometer analysis software. Specific ADCC lysis was calculated as follows: % specific lysis = 100 − [(average live target cell count for antibody treated samples/average live target count for control samples) × 100].

### Complement-Dependent Cytotoxicity (CDC) Assay

Complement-dependent cytotoxicity assays were performed using a modification of a previously described procedure (29). ASPC-1 target cells were labeled with calcein AM and seeded at 5.0 × 10^3^ cells/well into black-walled 96-well plates as described above in the ADCC assay methodology. Cells were then treated with 0.5 or 5.0 µg/mL NEO-201 for 15 min at 37°C to opsonize the target cells, and then purified rabbit complement (MP Biomedicals, Santa Ana, CA, USA) was added to each well at a final dilution of 1:8. After incubation at 37°C for 30, 60, or 120 min, 1.67 µg/mL propidium iodide was added to each well, plates were imaged and analyzed using the Celigo Imaging Cytometer, and specific lysis was calculated as described above for ADCC activity.

### Xenograft Antitumor Assay

Tumors were established in 6-week-old female athymic NU/NU nude mice (Charles River Laboratories International, Wilmington, MA, USA) by implanting a suspension of 4.0 × 10^6^ CFPAC-1 tumor cells in 1× PBS subcutaneously in the right flank of the mice. Once tumors reached ~100 mm^3^ in size, mice were sorted by tumor volume and randomized into five groups (*n* = 10 animals). Mice were then injected intraperitoneally with vehicle alone (saline solution), human IgG1 (250 µg), or NEO-201 (100 and 250 µg) on days 13, 17, and 20 post implantation. Mice also received intraperitoneal injection of approximately 1.0 × 10^7^ human PBMCs activated with IL-2 (200 U/mL treated overnight in culture) on days 14, 18, and 21 as a source of immune effector cells. One group of mice was treated similarly with NEO-201 but did not receive human PBMCs. Tumors were measured with digital calipers every 2–3 days, and tumor volumes were calculated according to the formula (width^2^ × length)/2 = mm^3^, where width was the shorter of the two measurements. Mice were also weighed weekly as a gross measure of general health. Mice with tumor volumes >2,000 mm^3^ were sacrificed. These animal experiments were conducted at Biocon, Inc. (Rockville, MD, USA). All experiments were reviewed and approved by the Institutional Animal Care and Use Committee (IACUC) review board of Biocon, Inc.

### Biodistribution Analysis

The biodistribution study was evaluated in tumor-bearing mice using radiolabeled NEO-201 (by Comparative Biosciences, Sunnyvale, CA, USA) using a procedure described previously (17). Briefly, male and female athymic NU/NU nude mice (Charles River Laboratories International, Wilmington, MA, USA) were injected subcutaneously in the flank with a 200 µL suspension of 4.0 × 10^6^ CFPAC-1 cells in 1× PBS. On day 14 after engraftment, mice were injected intravenously with 20 μCi of ^125^I-labeled NEO-201 and then necropsied after 1, 2, 4, or 7 days. Blood, tumor tissue, and internal organs (lungs, kidneys, liver, spleen, pancreas, intestines, and stomach) were harvested at each time point (*n* = 4 animals), all tissues were weighed, and radioactivity in tissues was measured using a gamma counter. Data for each mouse were first calculated as counts per minute/milligram tissue, and then tissue counts per minute values were normalized relative to blood counts per minute values. All experiments were reviewed and approved by the IACUC review board of Comparative Biosciences, Inc.

### Single-Dose Toxicity Study in Cynomolgus Monkeys

A single-dose toxicity study was conducted in purpose-bred cynomolgus monkeys to test NEO-201 for pharmacokinetics and toxicity after a single dose of NEO-201. The duration of the study was 15 days from dose administration, with an additional 14 days quarantine prior to dose administration to acclimate the monkeys to the study room. Eight male and eight female animals (two animals/sex/group) were dosed by slow intravenous infusion (approximately 30 ± 5 min infusion) of NEO-201 diluted in saline solution using an infusion pump and plastic disposable syringe with a catheter extension tubing at dose levels of 0, 5, 20, and 49 mg/kg, which was the highest attainable concentration of antibody. Blood samples were drawn in all animals that received NEO-201 at the following time points: pre-dose, 10 min, 1, 2, 4, 6, 24, 48, 72, 96, 168, and 336 h. Serum was prepared from the blood samples for pharmacokinetic and toxicology analysis. Whole blood was used for cellular analysis. NEO-201 levels in the serum were measured by ELISA using the Human Therapeutic IgG1 ELISA kit (Cayman Chemical, Ann Arbor, MI, USA) as per the manufacturer’s instructions. These animal experiments were conducted at SNBL USA, Ltd. (Everett, WA, USA). All experiments were reviewed and approved by the IACUC review board of SNBL USA, Ltd.

Laboratory tests included hematology and coagulation [baseline (BL), days 2, 8, 15]: CBC and differential, activated partial thromboplastin time, fibrinogen and prothrombin time; serum chemistry (BL, days 2, 8, 15): albumin, alkaline phosphatase, ALT, AST, total bilirubin, calcium, total cholesterol, creatine kinase, creatinine, glucose, inorganic phosphorus, total protein, triglyceride, sodium, potassium, chloride, globulin, albumin/globulin ratio, blood urea nitrogen (BUN); and urinalysis (BL, day 15): color, clarity, glucose, ketones, occult blood, protein, bilirubin, nitrites, pH, urobilinogen, leukocytes, volume, specific gravity; bioanalytical analysis (using ELISA)—(BL, 10 min, 1, 2, 4, 6, 24, 48, 72, 96, 168, and 336 h) from Groups 2 through 4 using Phoenix WinNonlin version 6.1 software (Certara USA, Princeton, NJ, USA). Animal body weight measurements were recorded (BL, days 7 and 14), and neutrophil counts were assessed (BL, days 2, 8, 15).

### Statistical Analysis

Data were analyzed using GraphPad Prism (GraphPad Software, La Jolla, CA, USA). Comparisons between two groups were conducted by *T*-test, and *p* < 0.05 was considered statistically significant. Comparisons between tumor growth curves were conducted by two-way ANOVA, and *p* < 0.05 was considered significant. Graphs depict the mean ± SD from one representative experiment performed in triplicate.

## Results

### NEO-201 Binds to Various Human Carcinoma Cell Lines

**Figure 1:**
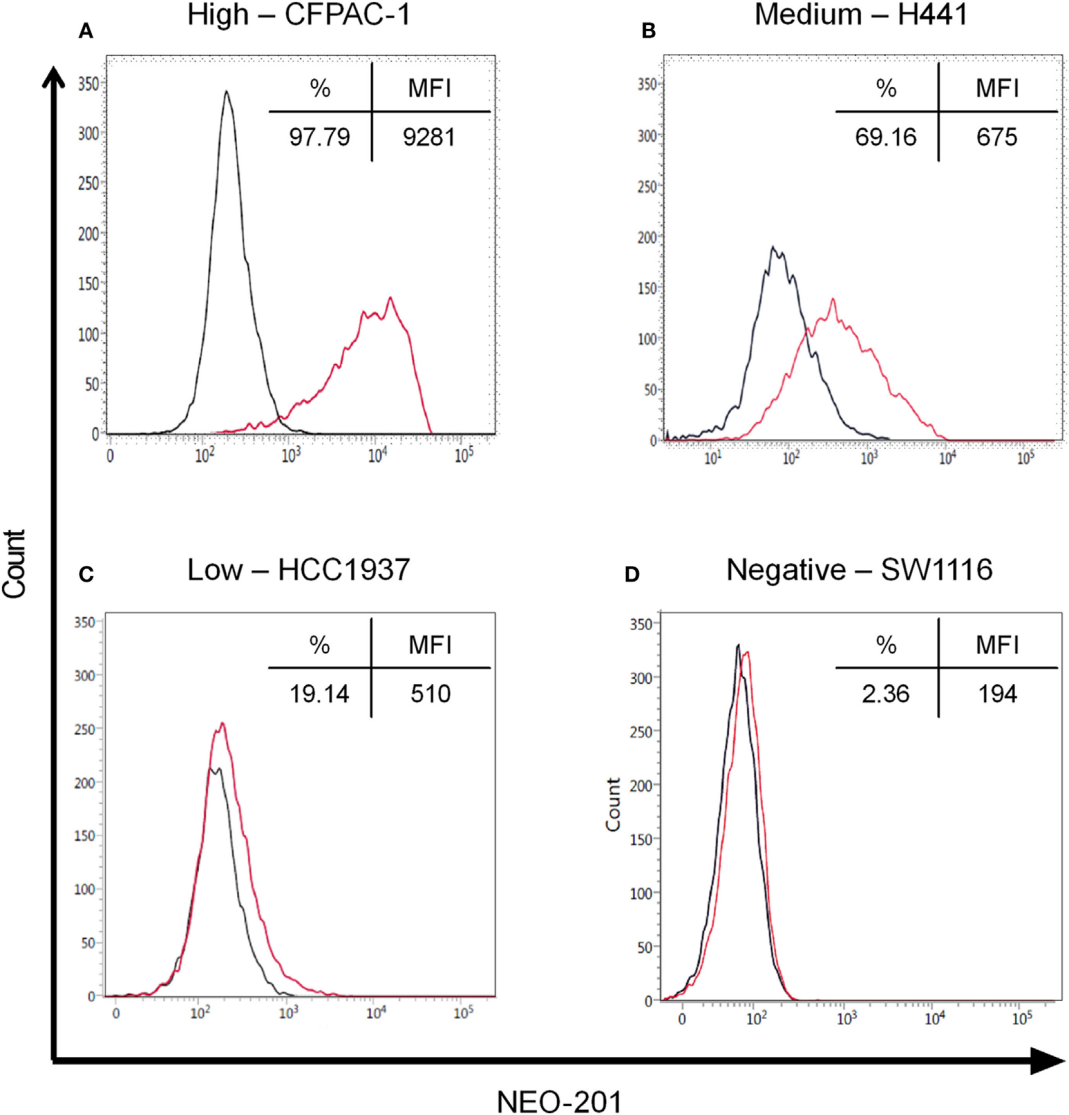
Flow cytometry of NEO-201 binding to human carcinoma cell lines. Representative human carcinoma cell lines with various levels of NEO-201 antigen expression, **(A)** pancreatic CFPAC-1 (high), **(B)** non-small cell lung carcinoma H441 (medium), **(C)** breast HCC1937 (low), and **(D)** colon SW1116 (negative). Results are expressed as % NEO-201 positive and median fluorescence intensity (MFI) for each cell line. Red, NEO-201-stained cells; black, unstained cells. NEO-201 positivity was defined as % positive ≥10%.

Flow cytometry analysis was used to profile a panel of human carcinoma cell lines for NEO-201 binding. The staining profile is summarized in Table 1, and representative histograms from cell lines with high, medium, low, and negative staining is shown in Figure 1. Assessment of the binding activity of NEO-201 revealed that 3/6 (50%) colon cancer cell lines and 4/5 (80%) pancreatic cancer cell lines were highly positive. When non-small cell lung carcinoma cell lines of various histological subtypes were profiled, it was determined that 3/5 (60%) of adenocarcinoma cell lines reacted with NEO-201, while only 1/4 (25%) of squamous cell carcinoma cell lines were found to be positive. Screening of breast cancer cell lines was also conducted. Of the cell lines that expressed either the estrogen receptor (ER) or the progesterone receptor (PR), whether alone or in combination with HER2, 2/4 (50%) stained positively for NEO-201. Of the HER2+ cell lines, whether alone or in combination with ER or PR, 3/4 (75%) were recognized by NEO-201. However, NEO-201 staining was found at low levels on only 1/4 (25%) of triple-negative breast cancer cell lines. In total, 15/30 (50%) of tested tumor cell lines were recognized by NEO-201. These data indicate that NEO-201 is reactive against a broad range of *in vitro* cultured tumor cell lines and show that distinct differences in antibody reactivity can occur based upon tumor subtype.

**Table 1:** Flow cytometry analysis of NEO-201 binding to tumor cell lines derived from various types of solid tumors.

Cell line	Tumor type	% Positive	MFI
**COLO 205**	**Colon**	**10.33**	**245**
**HT-29**	**Colon**	**38.40**	**352**
**LS174T**	**Colon**	**46.46**	**345**
SW1116	Colon	2.36	194
SW1463	Colon	1.23	278
SW480	Colon	1.70	575

**ASPC-1**	**Pancreatic**	**79.26**	**8,927**
**BxPC-3**	**Pancreatic**	**97.25**	**2,584**
**CAPAN-2**	**Pancreatic**	**29.69**	**327**
**CFPAC-1**	**Pancreatic**	**97.79**	**9,281**
PANC-1	Pancreatic	3.29	289

**H441**	**Non-small cell lung carcinoma (NSCLC) (adenocarcinoma)**	**69.16**	**675**
H522	NSCLC (adenocarcinoma)	1.38	238
**HCC4006**	**NSCLC (adenocarcinoma)**	**99.27**	**9,899**
**HCC827**	**NSCLC (adenocarcinoma)**	**77.46**	**692**
SK-LU-1	NSCLC (adenocarcinoma)	1.77	685

CALU-1	NSCLC (squamous)	4.22	571
H1703	NSCLC (squamous)	4.16	111
H226	NSCLC (squamous)	4.83	209
**H520**	**NSCLC (squamous)**	**61.78**	**443**

**AU-565**	**Breast (HER2+)**	**50.04**	**227**
**BT-474**	**Breast (PR+/HER2+)**	**68.79**	**591**
HCC1500	Breast (ER+/PR+)	1.53	597
SK-BR-3	Breast (HER2+)	1.61	329
T-47D	Breast (ER+/PR+)	8.00	161
**ZR-75-1**	**Breast (ER+/PR+/HER2+)**	**68.80**	**550**

BT-549	Breast (ER−/PR−/HER2−)	1.47	477
**HCC1937**	**Breast (ER−/PR−/HER2−)**	**19.14**	**510**
HCC38	Breast (ER−/PR−/HER2−)	2.15	226
MDA-MB-468	Breast (ER−/PR−/HER2−)	6.33	344

*The percentage of positive cells and median fluorescence intensity (MFI) values are detailed for each cell line. NEO-201 positive cell lines appear in bold text. NEO-201 positivity was defined as % positive >10%*.

### NEO-201 Tissue Staining Is Highly Tumor Specific

**Figure 2:**
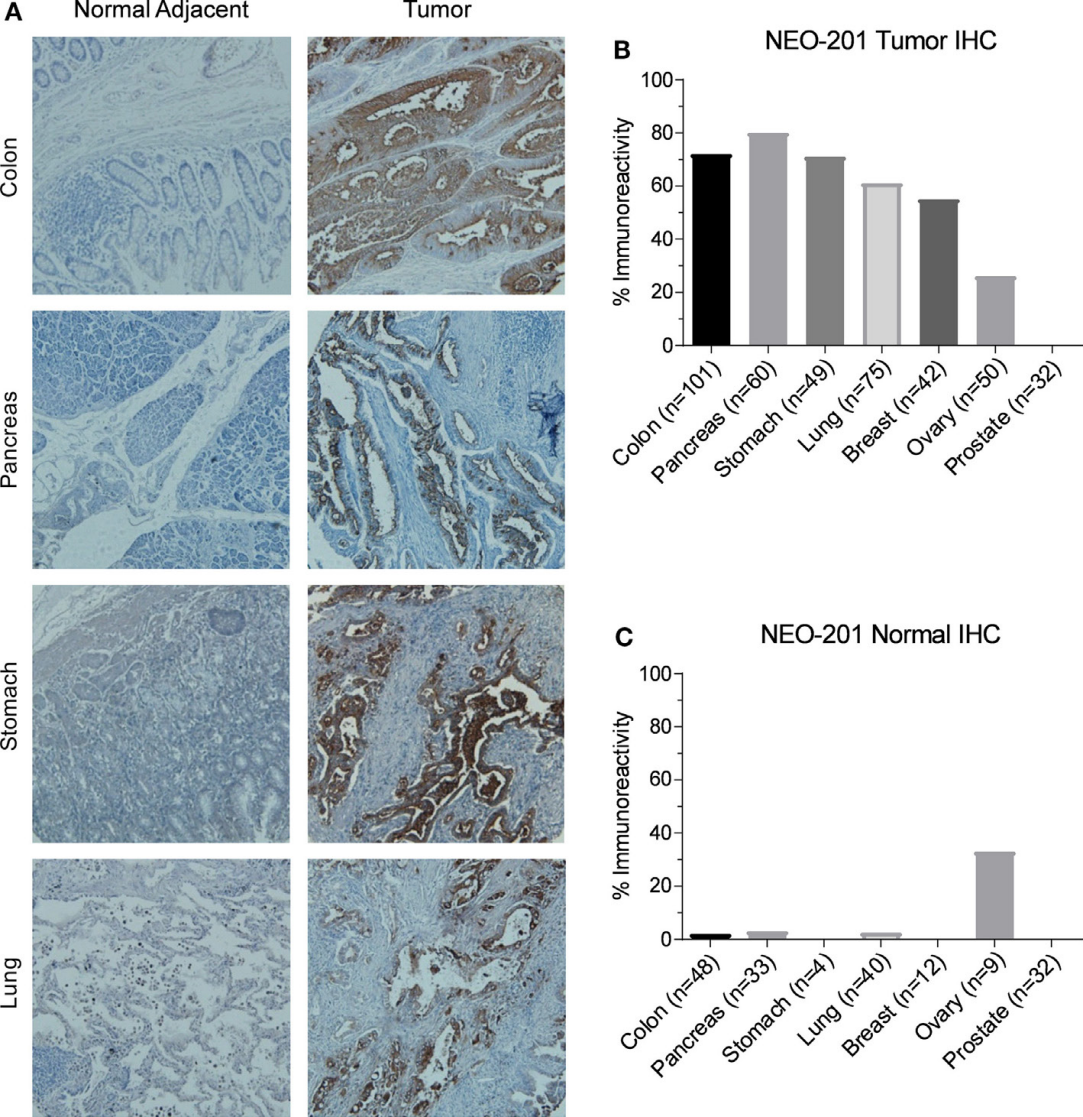
Immunohistochemistry (IHC) staining of human tumor samples by NEO-201. **(A)** Representative NEO-201 staining from normal and malignant tissues from colon, pancreas, stomach, and lung samples. All images were obtained at 100×. **(B)** Quantification of NEO-201 positive staining from the human tumor microarray samples from various carcinoma tissues. **(C)** Quantification of NEO-201 positive staining from normal tissue included in the human tumor microarray samples. *n*, number of samples.

Immunohistochemistry was used to investigate NEO-201 reactivity from human tumor samples using tissue microarrays representing dozens of samples for each cancer type. As shown in Figure 2A, immunoreactivity with NEO-201 was completely absent from normal colon, pancreas, stomach, and lung tissues, but was highly positive in the tumor tissues from these organs. Strikingly, staining was found only on the tumor cells, as the surrounding stromal cells were not stained (Figure 2A). IHC staining of the microarray samples determined that NEO-201 was highly reactive against colon cancer (72%), pancreatic cancer (80%), stomach cancer (71%), lung cancer (61%), and breast cancer (55%). Additionally, a sizeable minority of ovarian cancer (26%) samples also exhibited positive staining, but no staining was observed in prostate cancer tissues (Figure 2B). The strongest staining intensity was observed from the pancreatic and colon cancer samples (Figure S1 in Supplementary Material). Excluding prostate cancer, the overall positivity of sampled tumor tissues was 238/377 (63%). Importantly, NEO-201 reactivity was almost entirely absent from normal healthy tissues (Table 2), and from the normal tissues included in the tumor microarrays with the exception of some ovarian samples (Figure 2C). However, the number of tissues in this set of ovarian samples was limited (nine samples). Altogether, these data indicate that NEO-201 recognizes tumor tissues from a wide variety of carcinomas and is highly tumor-specific.

**Table 2:** Immunohistochemistry profile of NEO-201 staining of normal human microarray tissues.

Tissue type	Positive/total	Tissue type	Positive/total
Cerebral cortex	0/2	Spleen	0/2
Cerebellum	0/2	Lymph node	0/2
Basal ganglia	0/2	Tonsil	0/2
Hippocampus	0/2	Thymus	0/2
Spinal cord	0/2	Paratoid gland	0/2
Heart	0/2	Skeletal muscle	0/2
Lung	0/2	Ureter	0/2
Bronchus	0/2	Exocervix	2/2, weak
Tongue	2/2, weak	Endocervix	0/2
Esophagus	0/2	Pro-endometrium	0/2
Stomach	0/2	Sec-endometrium	0/2
Breast	0/2	Myometrium	0/2
Liver	0/2	Umbilical cord	0/2
Prostate	0/2	Soft tissue	0/2
Testis	0/2	Placenta; amnion	0/2
Ovary	0/2	Placenta; chorionvilli	0/2
Fallopian tube	0/2	Placenta; basal plate	0/2

### NEO-201 Mediates ADCC and CDC to Kill Tumor Cells

**Figure 3:**
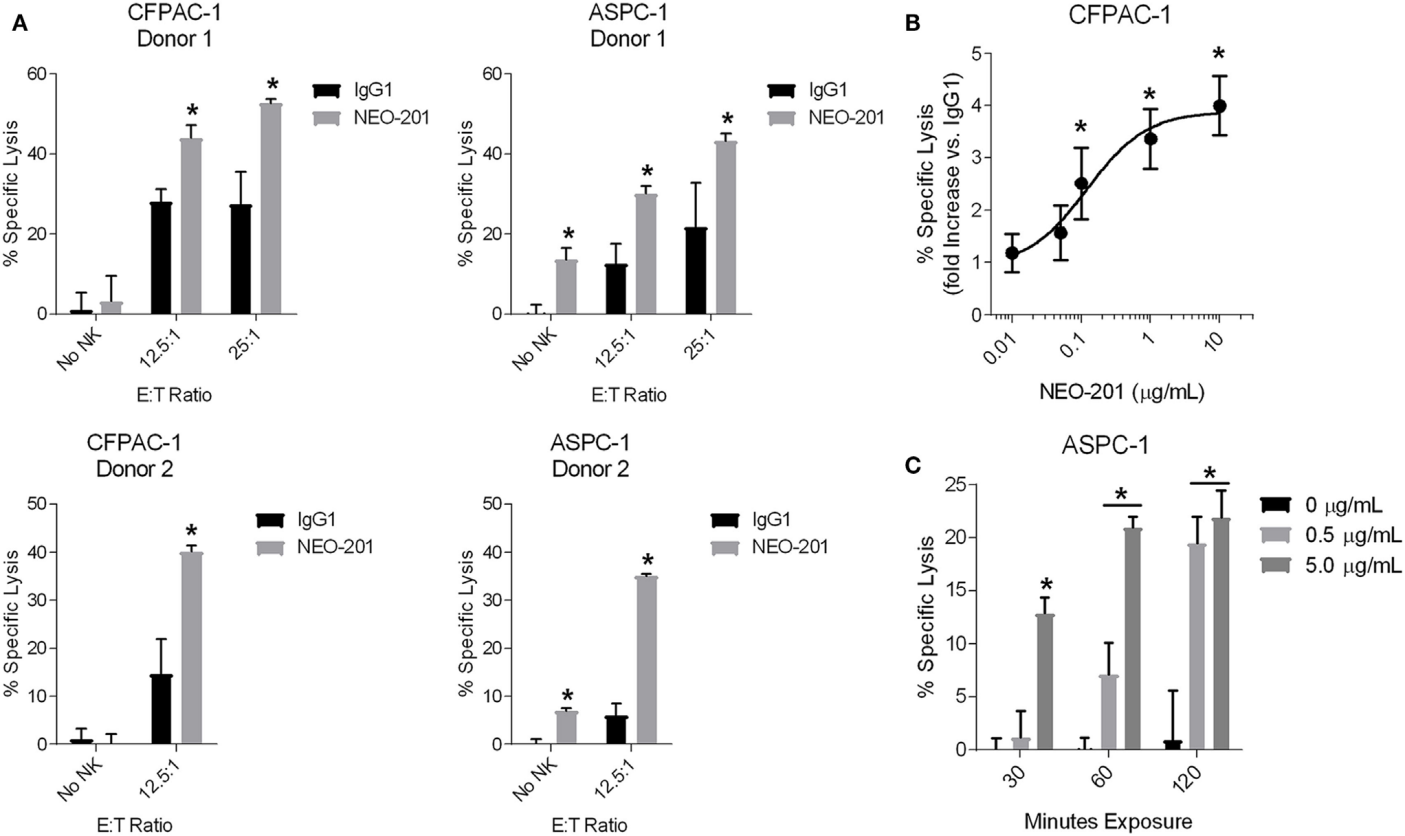
NEO-201 mediates ADCC and CDC against human tumor cell lines. **(A)** ADCC activity using CFPAC-1 or ASPC-1 cells as target cells. Cells were treated with 10 µg/mL of NEO-201 or human IgG1 (negative control). Purified natural killer (NK) cells from two healthy donors were used as effector cells at the indicated E:T ratios. *Statistically significant (*p* < 0.05) by *T*-test. **(B)** ADCC assay using CFPAC-1 cells treated with increasing doses of NEO-201. NK cells isolated from a healthy donor were used as effector cells at an E:T ratio of 12.5:1. The graph depicts the fold increase in % specific lysis of NEO-201-treated tumor cells versus that of control cells treated with 10 µg/mL human IgG1. *Statistically significant (*p* < 0.05) by *T*-test. **(C)** CDC assay using ASPC-1 cells treated with rabbit complement (1:8 dilution) and the indicated doses of NEO-201 for the indicated durations. *Statistically significant (*p* < 0.05) by *T*-test.

As a humanized IgG1 antibody, NEO-201 is theorized to be capable of mediating ADCC to kill tumor cells that express the NEO-201 antigen. To investigate this potential mechanism of action, ADCC assays utilizing human NK cells isolated from PBMCs from two different healthy donors were performed on cell lines highly positive for NEO-201 staining (CFPAC-1 and ASPC-1). Treatment with NEO-201 was observed to enhance the killing of both CFPAC-1 and ASPC-1 cells to levels two-fold to six-fold greater than the killing of control IgG1-treated tumor cells (Figure 3A). Titration assays were also conducted and revealed that NEO-201 retains the ability to significantly induce ADCC at doses as low as 0.1 µg/mL (Figure 3B).

Human IgG1 antibodies are also capable of mediating CDC, a complex cascade of proteolytic cleavages that culminates in the activation of the membrane attack complex that lyses antibody-bound target cells. CDC assays revealed that NEO-201 induces complement-mediated lysis of ASPC-1 cells in a manner that was dependent upon both mAb dose and incubation time (Figure 3C). Altogether, these data demonstrate that NEO-201 effectively engages innate immune effector mechanisms to specifically lyse antibody-bound tumor cells *in vitro*.

### NEO-201 Reduces the Growth of Tumor Xenografts Alone and in Combination with Human PBMC Effector Cells

**Figure 4:**
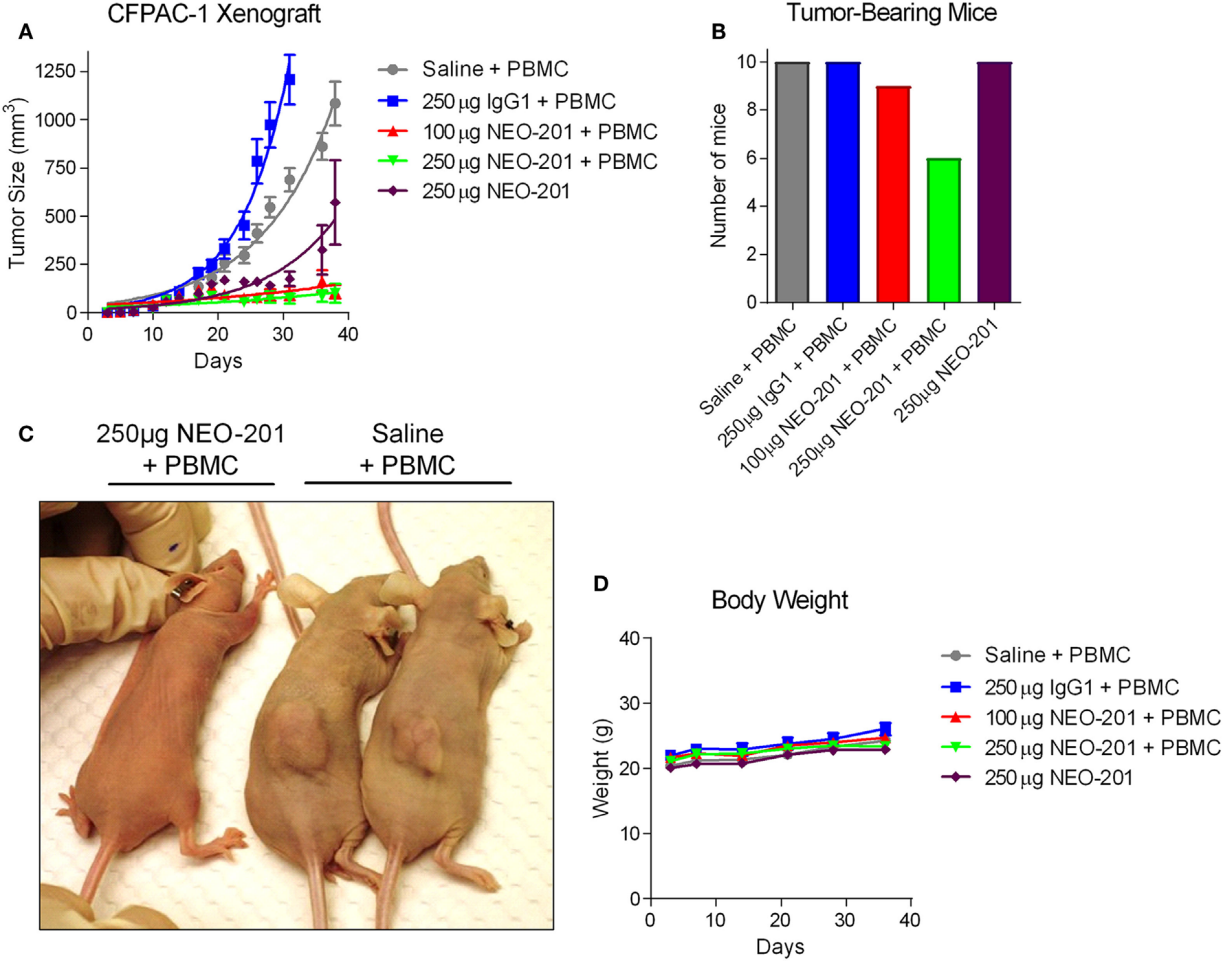
Antitumor efficacy of NEO-201 in CFPAC-1 tumor xenografts. **(A)** Tumor volume measurements for the CFPAC-1 xenografts from each treatment group at various time points. Mice (*n* = 10 animals/group) were dosed intraperitoneally with saline solution, human IgG1 (250 µg), or NEO-201 (100 and 250 µg) on days 13, 17, and 20 post-tumor cell implantation. Mice were also dosed intraperitoneally with ~1.0 × 10^7^ IL-2-activated human peripheral blood mononuclear cells (PBMCs) on days 14, 18, and 21 as a source of immune effector cells. **(B)** Quantification of the number of mice still bearing palpable tumors on day 36. **(C)** Representative image of NEO-201-treated versus saline-treated tumor-bearing mice. **(D)** Body weight measurements of the tumor-bearing mice at various time points during the study.

To determine the potential antitumor efficacy of NEO-201, CFPAC-1 cells were grown as tumor xenografts in immunocompromised NU/NU nude mice. These cells were chosen based upon their high expression level of NEO-201 antigen and high sensitivity to NEO-201-mediated ADCC. Once the CFPAC-1 tumors had grown to approximately 100 mm^3^ in size, tumor-bearing mice were injected three times with saline, 250 µg human IgG1, 100 µg NEO-201, or 250 µg NEO-201 followed by three injections of 1.0 × 10^7^ IL-2-activated (200 U/mL) human PBMCs to function as ADCC-mediating effector cells. As shown in Figure 4A, NEO-201 + PBMCs induced a substantial reduction in tumor growth at both dose levels compared to either the saline + PBMCs or human IgG + PBMCs control groups (*p* < 0.0001 by two-way ANOVA). Whereas no mice from the control groups were tumor-free on day 36, 1 of 10 (10%) and 4 of 10 (40%) mice had no palpable tumor remaining from the NEO-201 100 µg + PBMCs and the NEO-201 250 µg + PBMCs groups, respectively (Figure 4B). In addition, another group of mice were dosed with NEO-201 without the addition of human PBMCs, and a significant reduction in tumor growth relative to the control groups was observed (*p* < 0.0001 by two-way ANOVA; Figures 4A,C). Importantly, monitoring of the body weights of the tumor-bearing mice revealed no weight reduction in any of the treatment groups (Figure 4D). Collectively, these results indicate that NEO-201 is capable of substantially reducing tumor growth though both ADCC and non-ADCC mechanisms without inducing significant toxicity in mice.

### NEO-201 Localizes to the Xenograft Tumor Site

**Figure 5:**
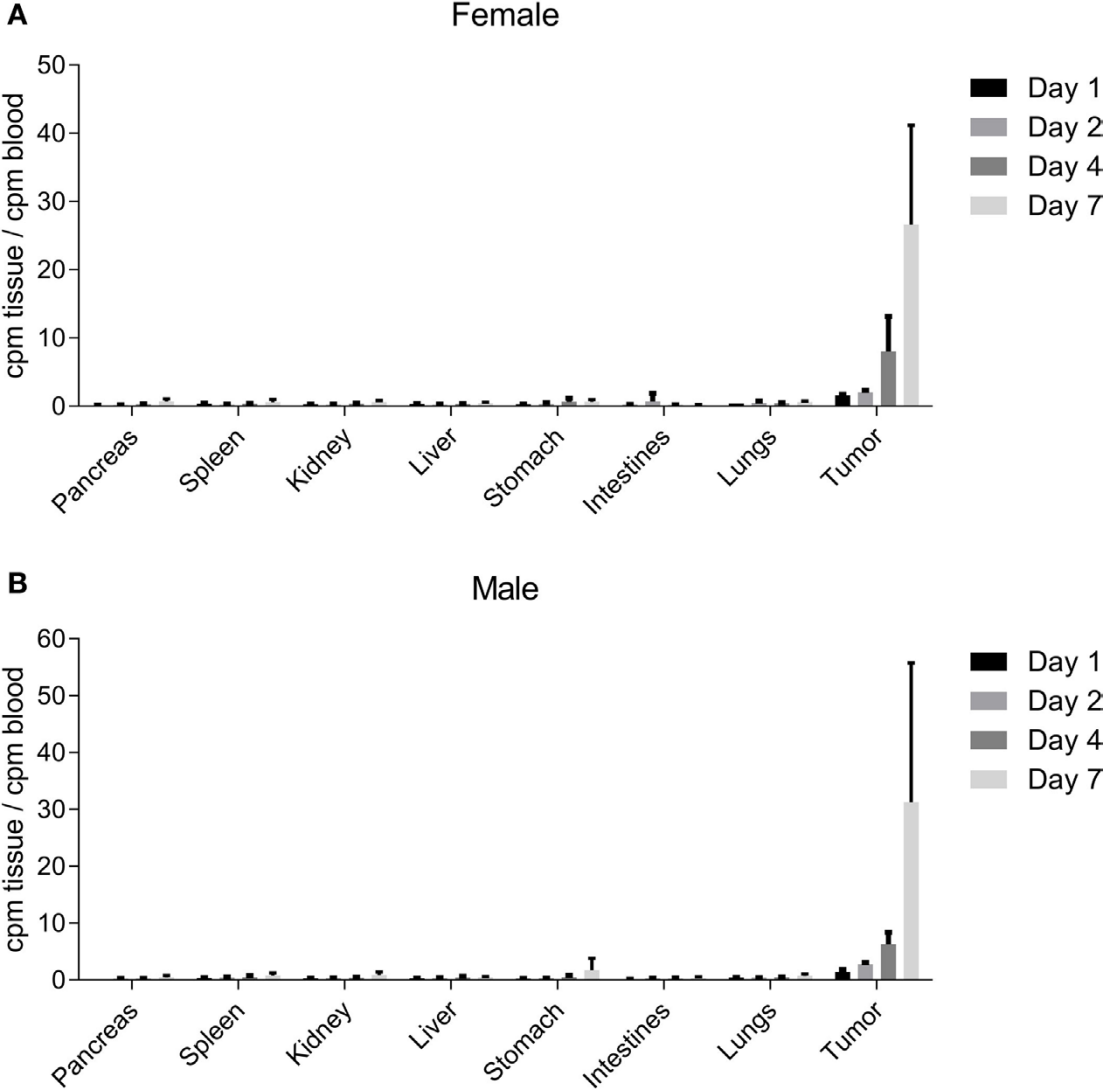
NEO-201 biodistribution in CFPAC-1 xenograft-bearing mice. Measurement of normalized radioactivity from the indicated tissues of CFPAC-1 tumor-bearing female **(A)** and male **(B)** mice dosed intravenously with radiolabeled NEO-201. *n* = 4 animals/time point. Days 1, 2, 4, and 7 represent the amount of time between radiolabeled antibody injection and necropsy.

Biodistribution studies were conducted utilizing radiolabeled NEO-201 in female and male NU/NU nude mice with established CFPAC-1 xenograft tumors. These mice were injected intravenously with the radiolabeled antibody, and then blood, organs, and tumors were harvested for analysis at various time points post-injection. Low levels of radioactivity were found in the pancreas, spleen, kidney, liver, stomach, intestines, and lungs in both male and female mice at all time points (Figures 5A,B). However, normalized uptake of radioactivity was substantially higher in tumors versus all other tissues at all time points, with tumor radioactivity progressively rising to levels 20–30 times higher than those of the blood by day 7 (Figures 5A,B). Quantitatively similar results were obtained for both female and male mice. These results indicate that NEO-201 preferentially localizes to malignant tissue that expresses the target antigen, and does not accumulate in normal tissues.

### NEO-201 Pharmacokinetics and Toxicity Evaluation in Non-Human Primates

A single-dose study was conducted in purpose-bred cynomolgus monkeys to determine NEO-201 pharmacokinetics and associated toxicity. Cynomolgus monkeys were selected because this species is closely related to humans both phylogenetically and physiologically and is a species commonly used for nonclinical toxicity evaluations. Male and female animals received a single intravenous infusion of NEO-201 diluted in saline at dose levels of 5, 20, and 49 mg/kg, which was the highest achievable dose per infusion volume. Blood samples were drawn in all animals pre-injection and at various time points post-injection up to 14 days, and serum preparations were assessed for NEO-201 levels by ELISA. As depicted in Table 3, quantifiable and dose-dependent serum concentrations of NEO-201 were observed through the last collection time point (14 days post-dose). As expected for an intravenous administration, Tmax values peaked by 10 min for the majority of the animals from all groups (10/12, 83%), with the exception of one male and one female animal each from the 5 mg/kg group. Over the dose range evaluated, peak (Cmax) exposure was dose proportional; total (AUC) exposure was greater than dose proportional at the lowest doses and approximately proportional from 20 to 49 mg/kg. Differences in exposure at the lowest dose were attributed to an approximately two-fold greater mean clearance (CL) and lesser volume of distribution (Vz). Mean half-life (HL) was 167 (20 mg/kg) or 170 (49 mg/kg) hours at the higher doses, approximately 3.7-fold greater than at the 5 mg/kg dose (46.2 h). Sex-differences were not observed.

**Table 3:** Pharmacokinetic results of single-dose NEO-201 administration in cynomolgus monkeys.

Dose level (mg/kg)	Sex	HL (h)	Tmax (h)	Cmax (μg/mL)	Cmax/D (μg/mL/mg)	AUCinf (h × μg/mL)	AUCinf/D (h × μg/mL/mg)	CL (mL/h)	Vz (mL)
5	M	58.5	0.584	135	10.4	8,210	640	1.67	137
	F	34.0	0.584	142	12.4	8,230	720	1.41	69.8
	All	46.2	0.584	138	11.4	8,220	680	1.54	103

20	M	176	0.167	639	12.3	77,600	1,500	0.669	171
	F	158	0.167	518	10.1	62,700	1,230	0.823	187
	All	167	0.167	579	11.2	70,100	1,360	0.746	179

49	M	122	0.167	1,460	11.6	126,000	1,000	1.00	174
	F	219	0.167	1,470	11.9	187,000	1,520	0.658	208
	All	170	0.167	1,470	11.8	157,000	1,260	0.830	191

*Eight male and eight female animals (two animals/sex/group) were injected intravenously with 0 (saline solution), 5, 20, or 49 mg/kg of NEO-201. Blood samples were drawn in all animals that received NEO-201 at various time points (pre-dose, 10 min, 1, 2, 4, 6, 24, 48, 72, 96, 168, and 336 h post-dose), and pharmacokinetic measurements from serum preparations were obtained by ELISA. Values in the table represent the average from the two animals/sex/group (M, F) or from all four animals (All)*.

*AUCinf, area under plasma concentration–time curve from time 0 to infinity; AUCinf/D, dose-normalized area under the plasma concentration–time curve from time 0 to infinity; CL, clearance; Cmax, maximum observed plasma concentration; Cmax/D, dose-normalized measured maximum plasma concentration; HL, half-life; Tmax, time of maximum observed plasma concentration; Vz, volume of distribution*.

**Figure 6:**
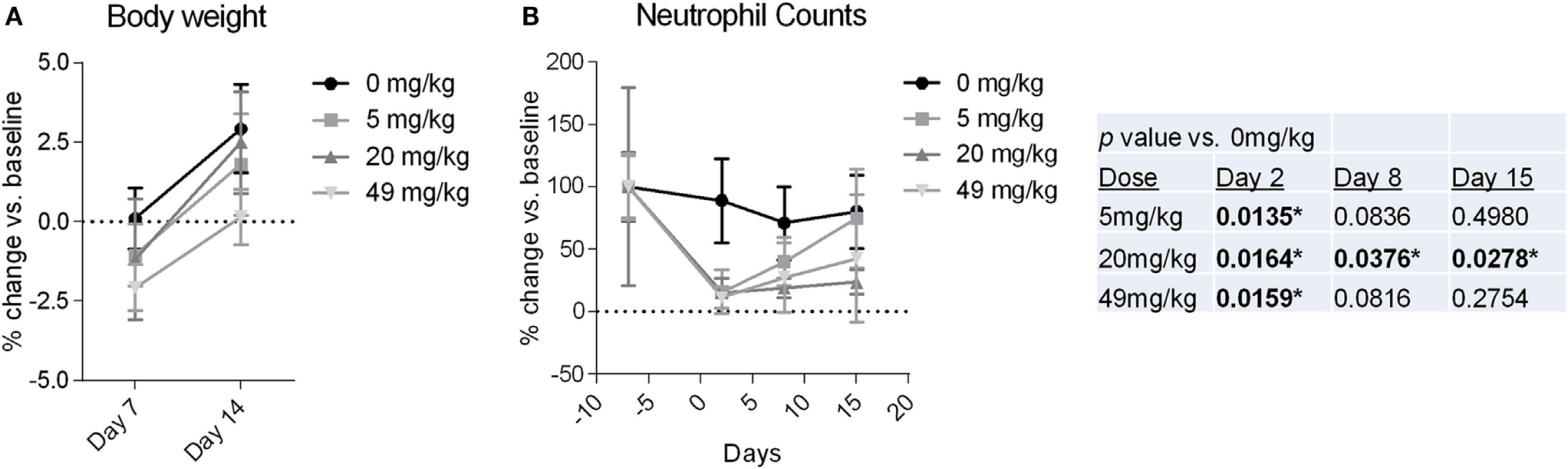
Body weight and neutrophil counts from cynomolgus monkeys treated with NEO-201. **(A)** Percent change in body weight relative to baseline (BL) (day −1) measured for monkeys at 7 and 14 days after a receiving a single dose of NEO-201 at the indicated dose levels. *n* = 4 animals per group (two females, two males). **(B)** Percent change in neutrophil levels relative to BL (day −7) from the blood of monkeys treated with a single dose of NEO-201 at the indicated dose levels. *n* = 4 animals per group (two females, two males). *Statistically significant (*p* < 0.05) by *T*-test.

Observations and examinations to determine toxicity over the course of the 14 day study included (1) periodic clinical evaluations; (2) measurement of food consumption and body weight; and (3) urine and blood evaluations, including urinalysis, hematology, coagulation tests, serum chemistry, and pharmacokinetics. As shown in Figure 6A, none of the dose level groups experienced a change in body weight >3% from their pre-injection weight, and no individual monkeys experienced a change >7%. Food consumption remained unchanged for all but two animals in the 5 mg/kg dose group who had low consumption on day 11 only. There were no significant changes from BL (before NEO-201 injection) through day 15 in any of the serum chemistry, urinalysis, or coagulation tests (see Materials and Methods for details). The main laboratory change in blood counts was a decrease in neutrophil counts relative to BL (Figure 6B). The decreases were of varying magnitudes, ranging from mild to marked, and a clear dose–response was not evident. For the majority of animals this was a transient finding, as improvements were typically noted by day 8 (Figure 6B). By day 15, neutrophil counts were observed to recover nearly totally or partially for the 5 mg/kg group or the 20 and 49 mg/kg groups, respectively (Figure 6B).

## Discussion

We have previously reported the preclinical antitumor activity (17) as well as clinical safety and efficacy (18, 19) for a mAb generated against the Hollinshead allogeneic colorectal cancer vaccine platform, termed ensituximab (NPC-1C/NEO-102). This report describes the characterization of the second novel tumor antigen-targeting mAb derived from the same vaccine platform, called NEO-201. NEO-201 was found to positively stain a variety of human carcinoma cell lines *in vitro*, including cells derived from a variety of tumor types, histological subtypes, and mutational profiles. NEO-201 positivity was more frequently observed in tumor cell lines derived from lung adenocarcinomas versus squamous cell carcinomas, and in HER2 positive breast cancer cell lines versus triple-negative lines. The staining of human tumor samples demonstrated that a wide variety of carcinoma tissues stained positively for NEO-201, including the colon, pancreatic, stomach, lung, and breast tumors. An expanded investigation with larger sample sizes may reveal that NEO-201 can discriminate between histological and/or molecular subtypes among various carcinomas. Intriguingly, a higher proportion of tumor tissues reacted with NEO-201 in contrast to cultured cancer cell lines. This observation may indicate that the target recognized by NEO-201 is expressed more readily *in vivo* than *in vitro*, which would suggest that target expression is at least partially dependent upon tumor cell interaction with factors from within the local microenvironment. Experiments are currently in progress to further characterize the antigen(s) and epitope(s) recognized by NEO-201 and to determine the regulatory control mechanism(s) which govern its expression in tumor tissue but not normal tissue.

This investigation also revealed that NEO-201 is remarkably tumor-specific in its staining profile, as the overwhelming majority of healthy normal tissues were found to be negative for NEO-201. Although NEO-201 positivity was observed in normal tongue and exocervix tissues, the staining intensity was weak and the microarray represented only a minimal sample size (*n* = 2). Further expanded analysis of NEO-201 staining in normal tissue samples will be undertaken to confirm these observations. Furthermore, NEO-201 administration did not induce any grossly observable toxicity in mice, and was well-tolerated when administered to non-human primates. The observed depletion of neutrophils in non-human primates suggests that the antigen(s) reactive with NEO-201 are expressed on these immune cells, and assessment of NEO-201 reactivity with hematopoietic cell types is ongoing. These encouraging results suggest that (1) NEO-201 may have diagnostic utility in discriminating cancerous from benign tissue from patient biopsies and (2) NEO-201 may effectively target tumors without provoking significant toxicity or off-target effects other than neutropenia. Efforts are currently underway to further evaluate the safety and tolerability of NEO-201, and a clinical trial using NEO-201 for the treatment of carcinoma is being planned.

Innate immune effector mechanisms have been shown to play a major role in promoting and potentiating host antitumor immunity. The Fc portion of human IgG1 mAbs is well known to activate innate immunity against opsonized targets by mediating both ADCC and CDC (30, 31). In particular, the ability to mediate ADCC is regarded as a key component of therapeutic efficacy for various human IgG1 mAbs approved for the treatment of cancer (21–27). Importantly, a V158F polymorphism in the *FCGR3A* gene (encoding FcγRIIIa) is associated with differential affinity for human IgG1 mAbs (32, 33), with immune cells from donors with the high affinity V/V genotype exhibiting greater trastuzumab-mediated ADCC activity *in vitro* (34). The V/V genotype was also shown to significantly correlate with objective response rate and progression-free survival in breast cancer patients treated with trastuzumab (34), thereby providing indirect clinical evidence for the role of ADCC in mAb-based therapy. NEO-201 can mediate ADCC *in vitro*, as treatment of tumor cells with NEO-201 enhanced the cytotoxic activity of NK cells by two-fold to five-fold, and ADCC activity was retained at even low concentrations of antibody (0.1 µg/mL). These data raise the possibility that patients with the V/V genotype may derive added benefit from NEO-201 treatment. An additional prospect is the potential to enhance ADCC activity, and presumably the potential clinical benefit of NEO-201, by augmenting NK cell function with cytokine stimulation. IL-2 is well known to be a potent activator of NK cells (35), and IL-21 was shown to enhance ADCC activity mediated by trastuzumab and cetuximab (36). Recent preclinical studies with a novel fusion protein superagonist of IL-15 signaling, termed ALT-803, have demonstrated greatly enhanced proliferation, activation, and lytic capability of NK cells (and CD8 + T cells), leading to significant antitumor activity in various animal models of cancer (37–42). Intriguingly, ALT-803 was found to substantially enhance *in vitro* NK cell degranulation, IFN-γ production, and rituximab-mediated ADCC against B cell lymphoma cell lines and primary follicular lymphoma cells, and combination treatment with ALT-803 and rituximab in two B cell lymphoma models *in vivo* resulted in significantly reduced tumor cell burden and improved survival (43). Future studies will evaluate the potential synergy between NEO-201 and novel immunotherapeutic reagents such as ALT-803.

Another innate immune effector mechanism engaged by mAbs is activation of the complement system to promote CDC, and NEO-201 was found to possess the ability to mediate CDC to kill tumor cells. The contribution of CDC to the therapeutic efficacy of mAbs is controversial but has been suggested to be beneficial for cancer therapy (28). Additionally, several different complement-regulatory proteins (CRPs) function to inhibit complement activation, and certain membrane-bound CRPs such as CD46, CD55, and CD59 were reported to be aberrantly expressed in various cancers (44–46), which likely confers resistance to CDC. Future investigations will ascertain whether strategies to block CRPs can enhance NEO-201-mediated CDC of resistant tumor cells.

Evaluation of NEO-201 *in vivo* revealed profound antitumor effects when dosed in combination with activated human immune effector cells. This combination even led to full regressions in some of the mice (5/20, 25%) from the two combination groups. Moreover, NEO-201 was found to preferentially localize to the xenograft tumor tissue but not to various healthy tissues. These data confirm that a crucial mechanism of action for NEO-201 against tumors is the ADCC-dependent lysis of tumor cells by innate immune cells. However, it should be noted that antitumor activity was also observed with NEO-201 alone without the addition of human immune cells to the immunodeficient mice. This phenomenon may be specific to conditions encountered *in vivo*, as treatment of CFPAC-1 tumor cells with NEO-201 did not induce substantial toxicity in the ADCC assays *in vitro*. One possible explanation for NEO-201 activity in the absence of immune effector cells may be the induction of CDC, as others have shown that complement depletion completely abrogated the antitumor effects of cetuximab in a human lung cancer cell line xenograft model (47). Experiments are currently underway to further determine any potential non-ADCC mechanisms (such as CDC) that may play a role in the *in vivo* antitumor activity of this antibody.

In summary, this investigation has demonstrated that NEO-201 is a remarkably tumor-specific antibody that is capable of engaging innate immune effector mechanisms to kill tumor cells. In addition, NEO-201 demonstrated safety and antitumor efficacy in an *in vivo* xenograft model of pancreatic cancer, as well as tolerability in non-human primates. These findings provide the supporting rationale for the clinical development of NEO-201 as a diagnostic and therapeutic agent for patients with a broad variety of carcinomas.

## Ethics Statement

All experimental studies involving animals were reviewed and approved by the Institutional Animal Care and Use committee (IACUC) of Biocon Inc., Comparative Biosciences, Inc., or SNBL USA, Ltd, respectively. All studies were conducted according to the Guide for the Care and Use of Laboratory Animals in facilities accredited by the Association for Assessment and Accreditation of Laboratory Animal Care (AAALAC).

## Author Contributions

MF, JD, AB, KT, and PA conceived and designed the research study. MF, JD, OS, AD, YC, SM, AB, KT, and PA developed the study methodology. MF, JD, OS, and AD acquired the data. MF, JD, OS, AD, YC, SM, AB, KT, and PA analyzed and interpreted the data. MF, JD, YC, SM, CA, AB, KT, and PA wrote, reviewed, and revised the manuscript. CA, KT, and PA provided administrative, technical, and material support. KT and PA supervised the study.

## Conflict of Interest Statement

MF, JD, OS, AD, YC, SM, KT, and PA conducted this research as employees of Precision Biologics, Inc. PA has ownership interest in Precision Biologics, Inc. AB and CA have no conflicts of interest to declare.

## Supplementary Material

Figure S1 NEO-201 immunohistochemistry staining intensities of human tumor samples. Quantification of NEO-201 positive staining intensities from the human tumor microarray samples from various carcinoma tissues. Sample staining intensity was scored on a scale of negative, 1+, 2+, 3+, and 4+. *n* = number of samples.

## References

[1] Ferlay J Soerjomataram I Dikshit R Eser S Mathers C Rebelo M Cancer incidence and mortality worldwide: sources, methods and major patterns in GLOBOCAN 2012. Int J Cancer (2015) 136(5):E359–86. 10.1002/ijc.29210 25220842

[2] Bodey B Siegel SE Kaiser HE. Human cancer detection and immunotherapy with conjugated and non-conjugated monoclonal antibodies. Anticancer Res (1996) 16(2):661–74. 8687112

[3] Mittal D Gubin MM Schreiber RD Smyth MJ New insights into cancer immunoediting and its three component phases – elimination, equilibrium and escape. Curr Opin Immunol (2014) 27:16–25. 10.1016/j.coi.2014.01.004 24531241 PMC4388310

[4] Dunn GP Old LJ Schreiber RD. The three Es of cancer immunoediting. Annu Rev Immunol (2004) 22:329–60. 10.1146/annurev.immunol.22.012703.104803 15032581

[5] Carter P. Improving the efficacy of antibody-based cancer therapies. Nat Rev Cancer (2001) 1(2):118–29. 10.1038/35101072 11905803

[6] Hodge JW Greiner JW Tsang KY Sabzevari H Kudo-Saito C Grosenbach DW Costimulatory molecules as adjuvants for immunotherapy. Front Biosci (2006) 11:788–803. 10.2741/1837 16146771

[7] Vergati M Intrivici C Huen NY Schlom J Tsang KY. Strategies for cancer vaccine development. J Biomed Biotechnol (2010) 2010:596432. 10.1155/2010/596432 20706612 PMC2914453

[8] Gabitzsch ES Tsang KY Palena C David JM Fantini M Kwilas A The generation and analyses of a novel combination of recombinant adenovirus vaccines targeting three tumor antigens as an immunotherapeutic. Oncotarget (2015) 6(31):31344–59. 10.18632/oncotarget.5181 26374823 PMC4741610

[9] Topalian SL Weiner GJ Pardoll DM. Cancer immunotherapy comes of age. J Clin Oncol (2011) 29(36):4828–36. 10.1200/JCO.2011.38.0899 22042955 PMC3255990

[10] Hollinshead A Glew D Bunnag B Gold P Herberman R Skin-reactive soluble antigen from intestinal cancer-cell-membranes and relationship to carcinoembryonic antigens. Lancet (1970) 1(7658):1191–5. 10.1016/S0140-6736(70)91784-8 4192376

[11] Hollinshead AC McWright CG Alford TGD Gold P Herbeman RB. Separation of skin reactive intestinal cancer antigen from the carcinoembryonic antigen of gold. Science (1972) 177(4052):887–9. 10.1126/science.177.4052.887 5054642

[12] Hollinshead A Elias EG Arlen M Buda B Mosley M Scherrer J. Specific active immunotherapy in patients with adenocarcinoma of the colon utilizing tumor-associated antigens (TAA). A phase I clinical trial. Cancer (1985) 56(3):480–9. 10.1002/1097-0142(19850801)56:3<480::AID-CNCR2820560312>3.0.CO;2-2 4005810

[13] Hollinshead AC Methods of Preparing Epitopes of Tumor Associated Antigens (1989). U.S. Patent No 4,810,781 A. Washington, DC: U.S. Patent and Trademark Office.

[14] Bristol JA Kantor JA Recombinant Monoclonal Antibodies and Corresponding Antigens for Colon and Pancreatic Cancers (2010). U.S. Patent No 7,829,678 B2. Washington, DC: U.S. Patent and Trademark Office.

[15] Hollinshead A. Active specific immunotherapy and immunochemotherapy in the treatment of lung and colon cancer. Semin Surg Oncol (1991) 7(4):199–210. 10.1002/ssu.2980070405 1925251

[16] Luka J Arlen PM Bristol A. Development of a serum biomarker assay that differentiates tumor-associated MUC5AC (NPC-1C ANTIGEN) from normal MUC5AC. J Biomed Biotechnol (2011) 2011:934757. 10.1155/2011/934757 21197415 PMC3010725

[17] Patel SP Bristol A Saric O Wang XP Dubeykovskiy A Arlen PM Anti-tumor activity of a novel monoclonal antibody, NPC-1C, optimized for recognition of tumor antigen MUC5AC variant in preclinical models. Cancer Immunol Immunother (2013) 62(6):1011–9. 10.1007/s00262-013-1420-z 23591984 PMC11029159

[18] Beg MS Azad NS Patel SP Torrealba J Mavroukakis S Beatson MA A phase 1 dose-escalation study of NEO-102 in patients with refractory colon and pancreatic cancer. Cancer Chemother Pharmacol (2016) 78(3):577–84. 10.1007/s00280-016-3108-5 27449137 PMC5238716

[19] Kim RD Arlen PM Tsang KY Mavroukakis SA Zaki A Cui K Ensituximab (E) in patients (pts) with refractory metastatic colorectal cancer (mCRC): Results of a phase 1/2 clinical trial. J Clin Oncol (2017) 35(Suppl):abstr3081 10.1200/JCO.2017.35.15_suppl.3081

[20] Zeligs K Arlen PM Tsang K Hernandez L Fantini M Annunziata CM Abstract 3025: Preclinical characterization of a novel monoclonal antibody targeting a neo-antigen expressed in ovarian and GI malignancies. Cancer Res (2017) 77(13 Suppl):3025 10.1158/1538-7445.AM2017-3025

[21] Seidel UJ Schlegel P Lang P. Natural killer cell mediated antibody-dependent cellular cytotoxicity in tumor immunotherapy with therapeutic antibodies. Front Immunol (2013) 4:76. 10.3389/fimmu.2013.00076 23543707 PMC3608903

[22] Petricevic B Laengle J Singer J Sachet M Fazekas J Steger G Trastuzumab mediates antibody-dependent cell-mediated cytotoxicity and phagocytosis to the same extent in both adjuvant and metastatic HER2/neu breast cancer patients. J Transl Med (2013) 11:307. 10.1186/1479-5876-11-307 24330813 PMC4029549

[23] Dall’Ozzo S Tartas S Paintaud G Cartron G Colombat P Bardos P Rituximab-dependent cytotoxicity by natural killer cells: influence of FCGR3A polymorphism on the concentration-effect relationship. Cancer Res (2004) 64(13):4664–9. 10.1158/0008-5472.CAN-03-2862 15231679

[24] Levy EM Sycz G Arriaga JM Barrio MM von Euw EM Morales SB Cetuximab-mediated cellular cytotoxicity is inhibited by HLA-E membrane expression in colon cancer cells. Innate Immun (2009) 15(2):91–100. 10.1177/1753425908101404 19318419

[25] Kawaguchi Y Kono K Mimura K Sugai H Akaike H Fujii H. Cetuximab induce antibody-dependent cellular cytotoxicity against EGFR-expressing esophageal squamous cell carcinoma. Int J Cancer (2007) 120(4):781–7. 10.1002/ijc.22370 17096332

[26] López-Albaitero A Lee SC Morgan S Grandis JR Gooding WE Ferrone S Role of polymorphic Fc gamma receptor IIIa and EGFR expression level in cetuximab mediated, NK cell dependent *in vitro* cytotoxicity of head and neck squamous cell carcinoma cells. Cancer Immunol Immunother (2009) 58(11):1853–64. 10.1007/s00262-009-0697-4 19319529 PMC3426289

[27] Boyerinas B Jochems C Fantini M Heery CR Gulley JL Tsang KY Antibody-dependent cellular cytotoxicity activity of a novel anti-PD-L1 antibody avelumab (MSB0010718C) on human tumor cells. Cancer Immunol Res (2015) 3(10):1148–57. 10.1158/2326-6066.CIR-15-0059 26014098 PMC4739754

[28] Meyer S Leusen JH Boross P. Regulation of complement and modulation of its activity in monoclonal antibody therapy of cancer. MAbs (2014) 6(5):1133–44. 10.4161/mabs.29670 25517299 PMC4622586

[29] Konishi E Kitai Y Kondo T. Utilization of complement-dependent cytotoxicity to measure low levels of antibodies: application to nonstructural protein 1 in a model of Japanese encephalitis virus. Clin Vaccine Immunol (2008) 15(1):88–94. 10.1128/CVI.00347-07 18032598 PMC2223849

[30] Strome SE Sausville EA Mann D. A mechanistic perspective of monoclonal antibodies in cancer therapy beyond target-related effects. Oncologist (2007) 12(9):1084–95. 10.1634/theoncologist.12-9-1084 17914078

[31] Hayes J Frostell A Karlsson R Müller S Millan-Martin S Pauers M Identification of Fc gamma receptor glycoforms that produce differential binding kinetics for rituximab. Mol Cell Proteomics (2017) 16(10):1770–88. 10.1074/mcp.M117.066944 28576848 PMC5629263

[32] Koene HR Kleijer M Algra J Roos D von dem Borne AE de Haas M. Fc gammaRIIIa-158V/F polymorphism influences the binding of IgG by natural killer cell Fc gammaRIIIa, independently of the Fc gammaRIIIa-48L/R/H phenotype. Blood (1997) 90(3):1109–14. 9242542

[33] Wu J Edberg JC Redecha PB Bansal V Guyre PM Coleman K A novel polymorphism of FcgammaRIIIa (CD16) alters receptor function and predisposes to autoimmune disease. J Clin Invest (1997) 100(5):1059–70. 10.1172/JCI119616 9276722 PMC508280

[34] Musolino A Naldi N Bortesi B Pezzuolo D Capelletti M Missale G Immunoglobulin G fragment C receptor polymorphisms and clinical efficacy of trastuzumab-based therapy in patients with HER-2/neu-positive metastatic breast cancer. J Clin Oncol (2008) 26(11):1789–96. 10.1200/JCO.2007.14.8957 18347005

[35] Hank JA Robinson RR Surfus J Mueller BM Reisfeld RA Cheung NK Augmentation of antibody dependent cell mediated cytotoxicity following *in vivo* therapy with recombinant interleukin 2. Cancer Res (1990) 50(17):5234–9. 2386933

[36] Watanabe M Kono K Kawaguchi Y Mizukami Y Mimura K Maruyama T Interleukin-21 can efficiently restore impaired antibody-dependent cell-mediated cytotoxicity in patients with oesophageal squamous cell carcinoma. Br J Cancer (2010) 102(3):520–9. 10.1038/sj.bjc.6605502 20029417 PMC2822939

[37] Han KP Zhu X Liu B Jeng E Kong L Yovandich JL IL-15:IL-15 receptor alpha superagonist complex: high-level co-expression in recombinant mammalian cells, purification and characterization. Cytokine (2011) 56(3):804–10. 10.1016/j.cyto.2011.09.028 22019703 PMC3221918

[38] Gomes-Giacoia E Miyake M Goodison S Sriharan A Zhang G You L Intravesical ALT-803 and BCG treatment reduces tumor burden in a carcinogen induced bladder cancer rat model; a role for cytokine production and NK cell expansion. PLoS One (2014) 9(6):e96705. 10.1371/journal.pone.0096705 24896845 PMC4045574

[39] Mathios D Park CK Marcus WD Alter S Rhode PR Jeng EK Therapeutic administration of IL-15 superagonist complex ALT-803 leads to long-term survival and durable antitumor immune response in a murine glioblastoma model. Int J Cancer (2016) 138(1):187–94. 10.1002/ijc.29686 26174883 PMC4696021

[40] Rhode PR Egan JO Xu W Hong H Webb GM Chen X Comparison of the superagonist complex, ALT-803, to IL15 as cancer immunotherapeutics in animal models. Cancer Immunol Res (2016) 4(1):49–60. 10.1158/2326-6066.CIR-15-0093-T 26511282 PMC4703482

[41] Kim PS Kwilas AR Xu W Alter S Jeng EK Wong HC IL-15 superagonist/IL-15RαSushi-Fc fusion complex (IL-15SA/IL-15RαSu-Fc; ALT-803) markedly enhances specific subpopulations of NK and memory CD8+ T cells, and mediates potent anti-tumor activity against murine breast and colon carcinomas. Oncotarget (2016) 7(13):16130–45. 10.18632/oncotarget.7470 26910920 PMC4941302

[42] Felices M Chu S Kodal B Bendzick L Ryan C Lenvik AJ IL-15 super-agonist (ALT-803) enhances natural killer (NK) cell function against ovarian cancer. Gynecol Oncol (2017) 145(3):453–61. 10.1016/j.ygyno.2017.02.028 28236454 PMC5447472

[43] Rosario M Liu B Kong L Collins LI Schneider SE Chen X The IL-15-based ALT-803 complex enhances FcγRIIIa-triggered NK cell responses and *in vivo* clearance of B cell lymphomas. Clin Cancer Res (2016) 22(3):596–608. 10.1158/1078-0432.CCR-15-1419 26423796 PMC4738096

[44] Seya T Matsumoto M Hara T Hatanaka M Masaoka T Akedo H. Distribution of C3-step regulatory proteins of the complement system, CD35 (CR1), CD46 (MCP), and CD55 (DAF), in hematological malignancies. Leuk Lymphoma (1994) 12(5–6):395–400. 10.3109/10428199409073780 7514063

[45] Niehans GA Cherwitz DL Staley NA Knapp DJ Dalmasso AP. Human carcinomas variably express the complement inhibitory proteins CD46 (membrane cofactor protein), CD55 (decay-accelerating factor), and CD59 (protectin). Am J Pathol (1996) 149(1):129–42. 8686736 PMC1865231

[46] Donin N Jurianz K Ziporen L Schultz S Kirschfink M Fishelson Z. Complement resistance of human carcinoma cells depends on membrane regulatory proteins, protein kinases and sialic acid. Clin Exp Immunol (2003) 131(2):254–63. 10.1046/j.1365-2249.2003.02066.x 12562385 PMC1808622

[47] Hsu YF Ajona D Corrales L Lopez-Picazo JM Gurpide A Montuenga LM Complement activation mediates cetuximab inhibition of non-small cell lung cancer tumor growth *in vivo*. Mol Cancer (2010) 9:139. 10.1186/1476-4598-9-139 20529262 PMC2893457

