# Supplementary figures and images for: Preclinical Characterization of a Novel Monoclonal Antibody NEO-201 for the Treatment of Human Carcinomas
Source: Front Immunol. 2018 Jan 4;8:1899. doi: 10.3389/fimmu.2017.01899 (PMC5758533; doi:10.3389/fimmu.2017.01899)

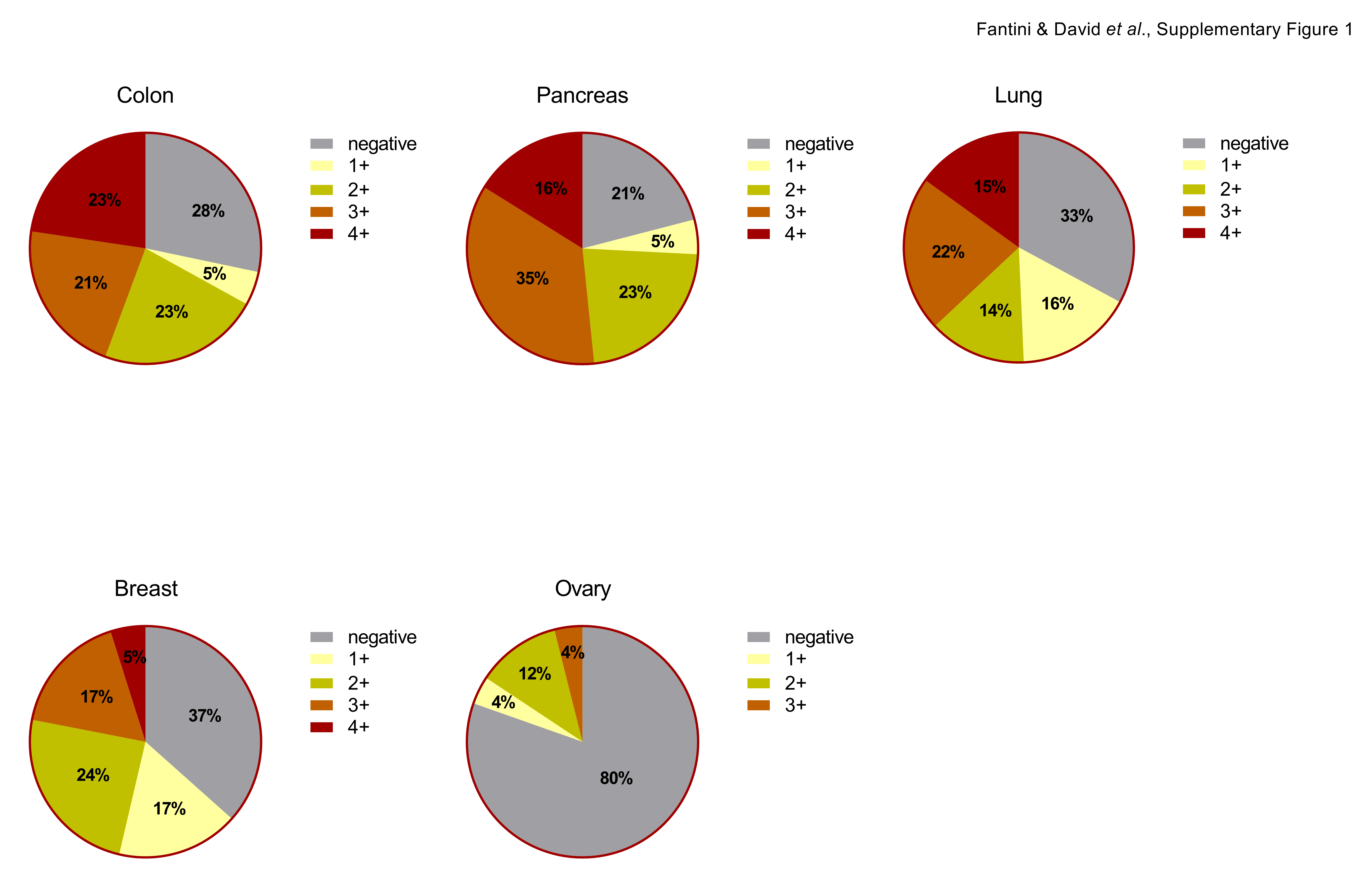

Supplement: Figure S1 — NEO-201 immunohistochemistry staining intensities of human tumor samples. Quantification of NEO-201 positive staining intensities from the human tumor microarray samples from various carcinoma tissues. Sample staining intensity was scored on a scale of negative, 1+, 2+, 3+, and 4+. n = number of samples. [file image_1.tif]
